# PARSEbp: pairwise agreement-based RNA scoring with emphasis on base pairings

**DOI:** 10.1093/bioadv/vbag112

**Published:** 2026-04-16

**Authors:** Sumit Tarafder, Debswapna Bhattacharya

**Affiliations:** Department of Computer Science, Virginia Tech, Blacksburg, VA 24061, United States; Department of Computer Science, Virginia Tech, Blacksburg, VA 24061, United States

## Abstract

**Motivation:**

High-fidelity scoring of RNA three-dimensional structures remains a major challenge in RNA structure prediction and conformational sampling. While single-model methods for scoring RNA structures can capture individual structural features, they fail to capture the broader structural consensus within a conformational ensemble, limiting their effectiveness in ranking and model selection.

**Results:**

We present PARSEbp, a fast and effective multi-model RNA scoring method that integrates pairwise structural agreement across the conformational ensemble with base pairing consistency. By leveraging both alignment-based global structural agreement at the three-dimensional level and base pairing consistency at the two-dimensional level, PARSEbp efficiently constructs a consensus similarity matrix from which per-structure accuracy scores are computed. Tested on RNA targets from the Critical Assessment of Structure Prediction (CASP) challenges CASP16 and CASP15, PARSEbp significantly outperforms existing single- and multi-model RNA scoring functions, including traditional statistical potentials, state-of-the-art deep learning methods, and consensus-based approaches, as well as a baseline variant of PARSEbp without the emphasis on base pairings, across a wide range of complementary assessment metrics.

**Availability and implementation:**

PARSEbp is freely available at https://github.com/Bhattacharya-Lab/PARSEbp.

## 1 Introduction

The three-dimensional (3D) structure of RNA plays a central role in determining its catalytic activity, regulatory capacity, and molecular interactions (Doudna and Cech 2002, Ganser *et al.* 2019). However, unlike proteins, RNAs are structurally flexible and can adopt diverse conformations stabilized by canonical and non-canonical base pairs, long-range interactions, and interactions with other molecules (Hohng *et al.* 2004, Schroeder *et al.* 2004, Vicens and Kieft 2022, Spitale and Incarnato 2023). Due to the inherent flexibility of RNA molecules, ensemble-based approaches for RNA 3D structure prediction are crucial to capture the range of conformations plausible for a given sequence (Laine *et al.* 2025). Recent advances in computational modeling of RNA 3D structures involving both deep learning-based methods (Li *et al.* 2023, Wang *et al.* 2023, Abramson *et al.* 2024, Tarafder and Bhattacharya 2025) and stochastic energy- or template-based approaches (Biesiada *et al.* 2016, Watkins *et al.* 2020) enable the generation of a large pool of candidate 3D structures for a given sequence. Despite the progress, automated prediction of RNA 3D structures still lags behind the human-expert modeling groups in the recent community-wide blind assessments (Miao *et al.* 2020, Das *et al.* 2023, Kretsch *et al.* 2025), largely due to data scarcity, structural complexity, and weak evolutionary signal of RNA sequences (Schneider *et al.* 2023, Tarafder *et al.* 2024). To render the computational ensembles practically useful, RNA scoring methods are essential to identify high-quality models from misfolded alternatives present in the ensemble to improve the accuracy of RNA 3D structure prediction (Mukherjee *et al.* 2024, Martinović *et al.* 2025).

Existing approaches for 3D RNA scoring can be broadly divided into two categories: single-model and multi-model methods. Single-model scoring methods evaluate each candidate structure independently without considering the alternative conformations available in the ensemble. These methods rely on either knowledge-based statistical potentials (Capriotti *et al.* 2011, Zhang *et al.* 2020, Tan *et al.* 2022, 2023, Lou *et al.* 2025) or deep learning architectures (Li *et al.* 2018, Townshend *et al.* 2021, Tarafder and Bhattacharya 2024, Liu *et al.* 2026) to estimate specific structural quality scores. However, all single-model scoring methods rely on experimental structures, either as a supervised signal to train their architecture or fine-tune the statistical potential parameters, thus limited to the paucity of the available RNA 3D structures in Protein Data Bank (PDB) (Berman *et al.* 2000). Additionally, such methods often fail to capture the broader consensus within a structure ensemble, limiting their effectiveness in ranking and model selection. In contrast, multi-model scoring methods can exploit the agreement across the ensemble of candidate structures to infer individual model quality in an unsupervised manner, without the need for experimental structures to train or fine-tune the parameters. A recent method RNAtive (Pielesiak *et al.* 2025), the only existing publicly available multi-model RNA scoring method in the literature, derives consensus base pairs by identifying recurrent patterns across RNA 3D folds to assign individual quality scores. However, RNAtive does not perform an explicit all-vs.-all pairwise comparison across the ensemble, limiting the ability to capture the full spectrum of structural agreement. This motivates the need to develop an accurate and computationally efficient multi-model RNA scoring method that can integrate both pairwise global structural similarity and base pairing fidelity across the entire ensemble to deliver robust quality estimates.

In this work, we present PARSEbp, a pairwise multi-model RNA scoring method that integrates global 3D structural similarity with two-dimensional (2D) base pairing consistency. PARSEbp computes pairwise TM-scores (Zhang *et al.* 2022) across the structural ensemble to capture global fold similarity with additional emphasis from pairwise Interaction Network Fidelity (INF) scores (Parisien *et al.* 2009), which quantifies the agreement of canonical and non-canonical base pairs between structures. These measures are combined into a consensus similarity matrix, from which per-structure quality scores are computed by averaging the agreement with all other models in the structural ensemble. Benchmarking on 25 RNA targets from the recently concluded CASP16 challenge, PARSEbp achieves the highest global Spearman’s correlation (0.92) while attaining the lowest top-1 loss of 0.05, demonstrating its effectiveness in top model selection from the diverse structural ensemble in CASP16. By unifying pairwise agreement at the 3D and 2D levels, PARSEbp provides a fast, accurate, and consensus-driven quality estimation tool, requiring only a few minutes to score an entire structural ensemble for a typical RNA of 100 nucleotides.

## 2 Materials and methods

Given a set of *N* RNA 3D structures $S=\{S_{1},S_{2},\ldots ,S_{N}\}$ as input, we evaluate the structural quality of the decoys by combining pairwise 3D structural similarity with base pair (2D) consistency among the structures in the pool. Specifically, for each ordered pair of structures $\left(S_{i},S_{j}\right)$ with i≠j, we compute the pairwise similarity score $T_{ij}$ as follows:


(1) $$T_{ij}=\text{TM‐score}(S_{i},S_{j})\;\forall i,j\;T_{ij}\in [0,1]$$

This results in a complete N×N pairwise similarity matrix $T=[T_{ij}]$ which encodes the global structural similarity in terms of TM-score (Zhang *et al.* 2022) across the ensemble. To guide this global pairwise similarity, we further assess the base pairing consistency among the structures by extracting 2D base pair map $S_{i}^{bp}$ from each 3D structure using MC-annotate (Gendron *et al.* 2001), a widely used method for RNA base pair annotation. For every pair of structures $\left(S_{i},S_{j}\right)$, we compute the pairwise Interaction Network Fidelity (INF) score (Parisien *et al.* 2009), which quantitatively measures the overlap of base pairing patterns as a degree of agreement between the two structures. INF is defined as the geometric mean between precision and recall and is calculated in the following way:


(2) $$\text{INF}=\sqrt{\frac{\left|TP\right|}{\left|TP|+|FP\right|}\times \frac{\left|TP\right|}{\left|TP|+|FN\right|}}$$

Here, true positives (*TP*) denote overlapping interaction pairs between structures $\left(S_{i}\right)$ and $\left(S_{j}\right)$ while false positives (*FP*) and false negatives (*FN*) denote the set of interactions only found in either $\left(S_{i}\right)$ or $\left(S_{j}\right)$, respectively. The pairwise base pair consistency $I_{ij}$ between two maps $S_{i}^{bp}$ and $S_{j}^{bp}$, yielding the pairwise base pair similarity matrix $I=[I_{ij}]$ is defined as:


(3) $$I_{ij}=\text{INF}(S_{i}^{bp},S_{j}^{bp})\;\forall i,j\;I_{ij}\in [0,1]$$

We combine the pairwise 3D and 2D agreements by taking an element-wise multiplication between the two similarity matrices T and I as follows:


(4) $$M=T\;\circ I,\;M_{ij}=T_{ij}I_{ij}$$

This yields the consensus matrix $M=[M_{ij}]$, which captures both the global 3D similarity between structures and the extent to which this similarity is reinforced by consistent base pairing interactions. The quality score $Q_{i}$ for each structure $S_{i}$ is defined as the mean agreement with all other structures in the ensemble:


(5) $$Q_{i}=\frac{1}{N-1}\sum_{\begin{array}{c} j=1\\ j\neq i \end{array}}^{N}M_{ij}$$

The resulting set of scores $Q=\{Q_{1},Q_{2},\ldots ,Q_{N}\}$ provides a quantitative self-assessment of the structures, where higher scores correspond to structures consistently supported by both 3D structural similarity and 2D base pairing patterns across the ensemble. To ensure scalability for long RNA sequences and large conformational ensembles, all pairwise computations are parallelized and executed in batches.

We evaluate our method PARSEbp against a broad set of both single-model and multi-model RNA scoring methods. Among the single-model methods, we compare against traditional knowledge-based statistical potentials such as rsRNASP (Tan *et al.* 2022), rsRNASP1 (Lou *et al.* 2025), cgRNAsp (Tan *et al.* 2023), DFIRE-RNA (Zhang *et al.* 2020), and RASP (Capriotti *et al.* 2011). rsRNASP, rsRNASP1, DFIRE-RNA, and RASP are all-atom statistical potentials, primarily scoring RNA 3D structures using distance-dependent pairwise interactions, with rsRNASP1 additionally incorporating torsion-angle (dihedral) features of the backbone, sugar, and bases, while RASP uses geometric descriptors to explicitly account for base-pairing and base-stacking interactions. In contrast, cgRNASP provides a coarse-grained alternative, using a reduced 3-bead (P, C4${\;}^{\prime }$, N) representation to score RNA 3D structures. Comparison against state-of-the-art deep learning-based approaches include RNA3DCNN (Li *et al.* 2018), ARES (Townshend *et al.* 2021), lociPARSE (Tarafder and Bhattacharya 2024), and RNArank (Liu *et al.* 2026). ARES and RNA3DCNN predict RMSD-based quality scores using SE(3)-equivariant graph convolution and a voxelized 3D density-grid representation of the RNA structure, respectively, with RNA3DCNN providing pretrained variants trained on samples generated from molecular dynamics (MD) simulation alone or on a combined MD and Monte Carlo (MC) dataset. In contrast, lociPARSE and RNArank are superposition-free lDDT-based approaches that predict per-nucleotide lDDT scores (pLDDT), which can be aggregated to obtain a global quality estimate. For multi-model methods, we compare PARSEbp against the only available web-based tool RNAtive (Pielesiak *et al.* 2025). RNAtive integrates optional user-specified 2D structural constraints and a choice of base pair analyzers prior to consensus ranking, where scoring is performed by comparing each structure against a consensus 2D interaction pattern inferred from the input structural ensemble. All methods are run locally using their default parameters. For predictions using RNA3DCNN, we use the model trained on samples from both MD and MC simulations. For RNAtive, we choose RNAView (Yang *et al.* 2003) as the base pair analyzer and use the “all” interaction score for evaluation. Additionally, we incorporate a baseline variant of our method PARSEbp without the emphasis on base-pairings to demonstrate the effectiveness of the emphasis on base-pairings.

Our benchmark set contains 1660 decoy 3D structures from 12 CASP15 targets and 4750 decoy 3D structures across 25 RNA targets from CASP16 with sequence length ≤500, as listed in Table 1, available as supplementary data at *Bioinformatics Advances* online. Targets with length >500 are omitted from evaluation, as CASP16 results indicate that they predominantly yielded low-quality decoys in the ensemble (Kretsch *et al.* 2025). Ground truth scores of all the structures are obtained from the official CASP16 assessments. Similarly, we obtain the ground truth scores from the official CASP15 assessment for all metrics, except the ground truth INF-All scores, which are missing from the CASP15 official assessment that we compute following (Eq. (2)) using FR3D (Sarver *et al.* 2008) annotations derived from the experimental and decoy structures. Following the CASP16 composite evaluation formula (Kretsch *et al.* 2025) proposed by the assessors, we define a composite score (*G*) as the same weighted combination of three structural similarity measures: TM-score (Zhang *et al.* 2022), Local Distance Difference Test (lDDT) (Mariani *et al.* 2013) with stereochemical checks enabled, and GDT_TS (Zemla 2003), as follows:


(6) G=0.3×TM-score+0.3×GDT_TS+0.4×lDDT

**Table 1:** Performance on 25 CASP16 RNA targets (4750 decoys) based on two separate metrics as ground-truth: composite score (*G*) and INF-All, sorted in non-decreasing order of global Pearson’s *r* for composite score (*G*) within each method type.^a^

Type	Method	Composite score (*G*)					INF-all				
		Global		Per-target average			Global		Per-target average		
		r↑	ρ↑	r↑	ρ↑	Loss ↓	r↑	ρ↑	r↑	ρ↑	Loss ↓
Single-model	RASP	0.43	0.37	0.55	0.59	0.24	0.60	0.47	0.72	0.68	0.10
	cgRNAsp	0.52	0.47	0.56	0.54	0.12	0.61	0.54	0.68	0.64	0.05
	ARES	0.52	0.51	0.57	0.57	0.21	0.65	0.62	0.72	0.66	0.10
	DFIRE-RNA	0.54	0.48	0.57	0.58	0.11	0.66	0.56	0.71	0.67	0.05
	rsRNAsp1	0.54	0.48	0.61	0.61	0.12	0.64	0.56	0.73	0.70	0.05
	RNA3DCNN	0.58	0.54	0.68	0.68	0.09	0.61	0.59	0.68	0.70	0.04
	rsRNAsp	0.60	0.55	0.62	0.61	0.15	0.64	0.56	0.73	0.70	0.09
	RNArank	0.65	0.68	0.66	0.59	0.10	0.57	0.62	0.61	0.55	0.06
	lociPARSE	0.69	0.67	0.70	0.67	0.09	0.77	0.74	0.79	0.73	0.04
Multi-model	RNAtive	0.50	0.60	0.59	0.69	0.13	0.59	0.65	0.75	**0.80**	0.13
	PARSEbp w/o bp^b^	0.84	0.83	0.83	0.79	**0.05**	0.78	0.78	0.79	0.73	**0.03**
	PARSEbp	**0.86**	**0.84**	**0.85**	**0.82**	**0.05**	**0.85**	**0.83**	**0.86**	**0.80**	**0.03**

a Values in bold indicate the best performance.

b A baseline variant of PARSEbp without the emphasis on base-pairings.

The scoring performance of various methods is evaluated using global Pearson’s correlation (*r*) and Spearman’s rank correlation (ρ) between predicted and ground truth scores, along with per-target average *r*, ρ, and loss (top-1 loss) where the metrics are computed first within each target, and then averaged across targets. Per-target loss is defined as the absolute difference between the ground truth of the top-ranked structure (ties, if any, are broken arbitrarily) and that of the best structure for each target. For consistency, we convert all predictions to a consistent convention where higher score denotes better quality, by negating outputs from methods that report energy- or RMSD-based fitness scores. Since global assessment aggregates decoy scores across targets before evaluation, it is important to normalize the raw score magnitudes, which can vary due to different length-scaling and decoy-pool difficulty. To ensure that the estimated decoy scores from all targets are comparable, we apply per-target min–max normalization to predicted scores from all methods and ground-truths, before pooling decoy scores across targets during global assessment. Consequently, all predicted quality scores and ground-truth metrics are mapped to [0,1] prior to performance evaluation.

## 3 Results

Table 1 summarizes the performance of PARSEbp against competing single-model and multi-model RNA scoring methods on 25 CASP16 RNA targets using the composite ground truth metric *G* (Eq. (6)) and INF-All. At the global level, PARSEbp achieves the highest Pearson’s correlation (r=0.86) and Spearman’s correlation (ρ=0.84) with respect to ground truth score *G*. On a per-target basis, our method consistently delivers superior correlations and the lowest average loss compared to all other methods. PARSEbp exhibits a remarkable performance advantage compared to single-model approaches. Knowledge-based statistical potentials such as rsRNASP, rsRNASP1, cgRNAsp, DFIRE-RNA, and RASP achieve only modest performance, with global correlations not exceeding r=0.60, underscoring the challenge of generalizing from limited reference states. Deep learning–based methods perform comparatively better, with lociPARSE attaining the highest global Pearson’s correlation (r=0.69) and highest per-target average Pearson’s correlation (r=0.70) among single-model approaches. In terms of per-target average loss, lociPARSE and RNA3DCNN both reach 0.09, representing the best performance within the single-model methods. Nonetheless, PARSEbp surpasses all single-model approaches in both global and per-target evaluations by large margins, achieving the highest per-target average Pearson’s correlation (r=0.85) and the lowest top-1 loss (0.05). Using INF-All as ground truth metric, PARSEbp again consistently achieves higher global and per-target correlations than the single-model methods as well as the lowest loss (0.03). This trend also holds on CASP15 (Table 2, available as supplementary data at *Bioinformatics Advances* online), where PARSEbp achieves a similarly low loss (0.04) and high correlations. To further assess whether PARSEbp remains effective when decoy pools are dominated by low-quality structures, we evaluate on the subset of CASP16 targets for which more than 50% of decoys are incorrectly folded (TM-score ≤0.45). Table 3, available as supplementary data at *Bioinformatics Advances* online, shows that, although the performance gap narrows on these challenging targets, PARSEbp still achieves the highest correlations among all the methods for both *G* and INF-All and attains the lowest top-1 loss for *G*, indicating robust ranking capability even with the abundance of low-quality structures. We further perform per-target average correlation computation using Fisher’s *z*-transformation and reported the results in Table 10, available as supplementary data at *Bioinformatics Advances* online. PARSEbp still remains the top-performing method across both composite metric *G* and INF-All. These results demonstrate the reliability of PARSEbp in consistently delivering top-notch performance across various complementary assessment metrics. Detailed per-target results of PARSEbp for CASP15 and CASP16 are available in Tables 4 and 5, available as supplementary data at *Bioinformatics Advances* online, respectively.

When considering multi-model RNA scoring approaches, PARSEbp is compared against RNAtive, the only other multi-model method currently available in the literature. As shown in Table 1, the performance of RNAtive remains modest with respect to ground truth *G*, with a per-target average loss of 0.13, higher than even the state-of-the-art single-model approaches. While RNAtive achieves competitive correlations when evaluated against INF-All, its top-1 loss remains much worse (0.13 vs. 0.03 for PARSEbp), making it less effective for selecting the best decoy as the top prediction from an ensemble of structures. To provide a more complete view of each method’s performance profile, we report additional assessments by individually considering various evaluation metrics, including lDDT, TM-score, RMSD, and GDT_TS as ground-truth in Tables 6–9, available as supplementary data at *Bioinformatics Advances* online, respectively. Across both CASP15 and CASP16, PARSEbp consistently attains the lowest top-1 loss for every metric, outperforming all other competing methods, including RNAtive, by selecting top-ranked structures that are consistently high-quality across multiple ground-truth criteria rather than any individual metric. To further assess the contribution of the emphasis on the base pair, we compare PARSEbp with a baseline variant that relies only on pairwise TM-score similarity, without the emphasis on base pairings. As shown in Table 1, removing base pairs leads to a noticeable drop in performance in terms of both global and per-target average correlations, particularly when INF-All is used as ground truth metric. The drops in correlations are statistically significant at 95% confidence level as revealed by the Wilcoxon signed-rank test with *P*-value =.02 for Pearson’s *r* and *P*-value =.03 for Spearman’s ρ, for the composite metric *G*, confirming that the incorporation of base pair consistency provides a meaningful gain in performance for multi-model RNA scoring. In summary, our method significantly outperforms the existing multi-model RNA scoring approach RNAtive and base pair information meaningfully contributes to the improved performance of PARSEbp.

In terms of computational efficiency, PARSEbp completes ensemble-wide scoring for typical RNA targets of length ∼100 nucleotides within half a minute. By comparison, RNAtive requires substantially longer runtime (approximately half an hour) even for shorter targets (≤100). For a concrete comparison, we generate an ensemble of 1000 sample 3D structures using RNAbpFlow (Tarafder and Bhattacharya 2025) for a CASP16 target R1255 of length 124 nucleotides and plot the time required to score the entire ensemble by each competing method in Figure 1, available as supplementary data at *Bioinformatics Advances* online. While lociPARSE (Tarafder and Bhattacharya 2024) is the fastest among single-model baselines, PARSEbp achieves runtime comparable to several single-model methods, despite being a multi-model approach relying on pairwise similarity evaluation. Therefore, PARSEbp is suitable to incorporate into large-scale prediction pipelines, trading modest additional runtime cost for its improved accuracy gain. In contrast, RNAtive is orders of magnitude slower on the same ensemble, highlighting the comparative suitability of PARSEbp for large-scale applications. Runtime analysis on the CASP16 benchmark set, provided in Figure 2, available as supplementary data at *Bioinformatics Advances* online, further confirms that PARSEbp exhibits scalability with increasing sequence length and structural ensemble size without sacrificing accuracy. Collectively, these results demonstrate that PARSEbp is a robust and accurate method for multi-model RNA scoring.

## 4 Conclusion

We present PARSEbp, a fast, accurate, and reliable multi-model RNA scoring method that unifies pairwise global structural similarity at the 3D level and base pairing consistency at the 2D level. By explicitly performing all-vs-all alignment-based comparisons given a structural ensemble with emphasis on base pairings, PARSEbp provides consensus-driven high-fidelity scoring of RNA 3D structures, significantly outperforming all existing single- and multi-model methods in CASP15 and CASP16 test sets. Despite its strengths, a key limitation of PARSEbp can be the all-versus-all pairwise comparison, which can substantially increase runtime for very large targets. Furthermore, due to the consensus-based nature of PARSEbp, its effectiveness may depend on the quality of the input structural ensemble or the presence of RNAs with multiple conformational states which are equally plausible (e.g. bistable RNA such as a riboswitch). Nonetheless, PARSEbp is computationally practical for large ensembles, with runtime comparable to several single-model baselines, and remains effective even when decoy pools are dominated by low-quality models, making it broadly useful for realistic downstream ensemble-scoring in RNA 3D structure prediction pipelines at scale.

## Supplementary Material

vbag112_Supplementary_Data

## Data Availability

The open-source PARSEbp method is freely available at https://github.com/Bhattacharya-Lab/PARSEbp. The RNA 3D decoy structures and corresponding ground truth scores for the CASP16 RNA targets have been downloaded from https://predictioncenter.org/download_area/CASP16/predictions/RNA/ and https://predictioncenter.org/casp16/results.cgi?view=targets&tr_type=rna, respectively. For CASP15, the 3D decoy structures and ground truth scores have been downloaded from https://predictioncenter.org/download_area/CASP15/predictions/RNA/ and https://predictioncenter.org/casp15/results.cgi? tr_type=rna, respectively.
