# Supplementary material for: PARSEbp: pairwise agreement-based RNA scoring with emphasis on base pairings
Source: Bioinform Adv. 2026 Apr 16;6(1):vbag112. doi: 10.1093/bioadv/vbag112 (PMC13132658; doi:10.1093/bioadv/vbag112)
Supplement: vbag112_Supplementary_Data [file vbag112_supplementary_data.pdf]

Supplementary Information for

PARSEbp: Pairwise Agreement-based RNA Scoring with Emphasis on Base Pairings

Sumit Tarafder[1] and Debswapna Bhattacharya[1*]

[1]Department of Computer Science, Virginia Tech, Blacksburg, Virginia, 24061, USA

Contents

1 Supplementary Tables **2**
1.1 List of targets in CASP15 and CASP16 benchmark set 2
1.2 Performance on CASP15 2
1.3 Performance of PARSEbp on challenging CASP16 decoy pools 3
1.4 Detailed per-target average results of PARSEbp in CASP15 3
1.5 Detailed per-target average results of PARSEbp in CASP16 4
1.6 Performance w.r.t. lDDT as ground truth 5
1.7 Performance w.r.t. TM-score as ground truth 5
1.8 Performance w.r.t. RMSD as ground truth 6
1.9 Performance w.r.t. GDT_TS as ground truth 6
1.10 CASP16 performance using Fisher-z averaged per-target correlations 7

2 Supplementary Figures **8**
2.1 Runtime comparison with SOTA methods 8
2.2 Runtime requirement of PARSEbp 9

*Corresponding Author. Email: dbhattacharya@vt.edu

1 Supplementary Tables

1.1 List of targets in CASP15 and CASP16 benchmark set

Table S1: Summary of CASP15 and CASP16 RNA targets and the number of decoy 3D structures.

CASP15			CASP16					
**ID**	**Length**	**# Decoys**	**ID**	**Length**	**# Decoys**	**ID**	**Length**	**# Decoys**
R1107	69	131	R1288	58	193	R1211	90	185
R1108	69	115	R1205	59	187	R1255	124	190
R1116	157	145	R1263	64	217	R1256	127	188
R1117	30	153	R1264	64	206	R1203	134	183
R1126	363	140	R1209	72	213	R1242	205	191
R1128	238	137	R1296	72	230	R1212	247	202
R1136	374	158	R1271	77	173	R1289	284	157
R1138	720	130	R1293	82	214	R1224s2	395	196
R1149	124	138	R1221s3	86	185	R1221s2	398	189
R1156	135	145	R1224s3	86	199	R1248	407	172
R1189	173	136	R1261	89	227	R1254	413	83
R1190	173	132	R1262	89	216	R1241	480	180
						R1291	480	174

1.2 Performance on CASP15

Table S2: Performance on 12 CASP15 RNA targets (1,660 decoys) based on two separate metrics as ground-truth: composite score (G) and INF-All, sorted in increasing order of global Pearson's r for composite score (G) within each method type. Values in bold indicate the best performance.

Type	Method	Composite score (G)					INF-All				
		Global		Per-target average			Global		Per-target average		
		$r \uparrow$	$\rho \uparrow$	$r \uparrow$	$\rho \uparrow$	Loss $\downarrow$	$r \uparrow$	$\rho \uparrow$	$r \uparrow$	$\rho \uparrow$	Loss $\downarrow$
Single-Model	ARES	0.36	0.31	0.55	0.51	0.16	0.58	0.59	0.57	0.53	0.22
	RNA3DCNN	0.37	0.44	0.41	0.37	0.14	0.37	0.35	0.41	0.41	0.13
	RASP	0.44	0.48	0.54	0.51	0.14	0.47	0.40	0.49	0.39	0.29
	RNArank	0.53	0.54	0.63	0.54	0.09	0.31	0.33	0.47	0.44	0.06
	rsRNAsp1	0.60	0.59	0.70	0.63	0.07	0.49	0.36	0.63	0.48	0.11
	cgRNAsp	0.62	0.61	0.62	0.48	0.10	0.40	0.27	0.53	0.33	0.20
	DFIRE-RNA	0.65	0.64	0.69	0.56	0.16	0.57	0.43	0.65	0.46	0.28
	lociPARSE	0.66	0.63	0.71	0.64	0.10	0.66	0.63	0.66	0.57	0.16
	rsRNAsp	0.73	0.74	0.70	0.60	0.10	0.42	0.32	0.59	0.44	0.19
Multi-Model	RNAtive	0.45	0.36	0.62	0.70	0.18	**0.85**	0.79	0.82	**0.82**	0.21
	PARSEbp	**0.74**	**0.72**	**0.85**	**0.85**	**0.05**	**0.85**	**0.80**	**0.88**	0.79	**0.04**

1.3 Performance of PARSEbp on challenging CASP16 decoy pools

Table S3: Performance on 12 CASP16 RNA targets that contain challenging decoy pools, defined as targets for which more than 50% of decoys are incorrectly folded (TM-score $\leq$ 0.45). Results are reported using two separate ground-truth metrics: composite score (G) and INF-All, sorted in increasing order of global Pearson's r for composite score (G) within each method type. Values in bold indicate the best performance.

Type	Method	Composite score (G)					INF-All				
		Global		Per-target average			Global		Per-target average		
		$r \uparrow$	$\rho \uparrow$	$r \uparrow$	$\rho \uparrow$	Loss $\downarrow$	$r \uparrow$	$\rho \uparrow$	$r \uparrow$	$\rho \uparrow$	Loss $\downarrow$
Single-Model	RNArank	0.43	0.40	0.51	0.46	0.11	0.36	0.32	0.44	0.36	0.07
	cgRNAsp	0.47	0.42	0.47	0.42	0.14	0.54	0.45	0.63	0.58	0.07
	ARES	0.48	0.48	0.52	0.55	0.11	0.60	0.56	0.69	0.66	0.08
	rsRNAsp1	0.51	0.45	0.53	0.52	0.12	0.58	0.51	0.69	0.66	0.06
	DFIRE-RNA	0.52	0.46	0.51	0.50	0.11	0.64	0.53	0.69	0.63	0.05
	RASP	0.52	0.48	0.57	0.58	0.12	0.68	0.52	0.76	0.69	0.07
	rsRNAsp	0.53	0.49	0.53	0.51	0.12	0.56	0.47	0.68	0.65	0.06
	lociPARSE	0.61	0.57	0.64	0.63	0.09	0.72	0.68	0.74	0.70	**0.04**
	RNA3DCNN	0.62	0.57	0.67	0.63	0.10	0.61	0.58	0.67	0.64	0.06
Multi-Model	RNAtive	0.34	0.42	0.46	0.59	0.17	0.48	0.48	0.66	**0.75**	0.17
	PARSEbp	**0.71**	**0.70**	**0.74**	**0.74**	**0.08**	**0.78**	**0.73**	**0.80**	0.74	0.05

1.4 Detailed per-target average results of PARSEbp in CASP15

Table S4: Per-target average performance of PARSEbp on 12 CASP15 RNA targets in terms of Pearson's correlation (r), Spearman's correlation (ρ), and top-1 loss w.r.t. composite score (G) as ground truth. Each row represents the average metrics for a target across its structural decoy ensemble.

ID	Pearson's r	Spearman's ρ	Top-1 Loss
R1107	0.79	0.74	0.12
R1108	0.89	0.91	0.10
R1116	0.95	0.92	0.07
R1117	0.82	0.83	0.01
R1126	0.85	0.92	0.01
R1128	0.84	0.82	0.00
R1136	0.96	0.92	0.02
R1138	0.90	0.95	0.01
R1149	0.93	0.81	0.08
R1156	0.90	0.85	0.18
R1189	0.71	0.79	0.01
R1190	0.71	0.75	0.02
**Average**	**0.85**	**0.85**	**0.05**

1.5 Detailed per-target average results of PARSEbp in CASP16

Table S5: Per-target average performance of PARSEbp on 25 CASP16 RNA targets in terms of Pearson's correlation (r), Spearman's correlation (ρ), and top-1 loss w.r.t. composite score (G) as ground truth. Each row represents the average metrics for every target across its own structural decoy ensemble.

ID	Pearson's r	Spearman's ρ	Top-1 Loss
R1288	0.58	0.66	0.05
R1205	0.74	0.72	0.02
R1263	0.93	0.96	0.01
R1264	0.93	0.95	0.00
R1209	0.67	0.77	0.00
R1296	0.79	0.83	0.15
R1271	0.93	0.83	0.02
R1293	0.68	0.71	0.21
R1221s3	0.91	0.79	0.06
R1224s3	0.92	0.85	0.05
R1261	0.95	0.88	0.05
R1262	0.95	0.88	0.05
R1211	0.82	0.77	0.19
R1255	0.85	0.87	0.01
R1256	0.76	0.77	0.05
R1203	0.93	0.93	0.02
R1242	0.93	0.90	0.03
R1212	0.80	0.83	0.10
R1289	0.97	0.93	0.01
R1224s2	0.98	0.95	0.02
R1221s2	0.96	0.92	0.05
R1248	0.86	0.86	0.05
R1254	0.43	0.25	0.03
R1241	0.98	0.88	0.05
R1291	0.98	0.83	0.07
**Average**	**0.85**	**0.82**	**0.05**

1.6 Performance w.r.t. lDDT as ground truth

Table S6: Performance comparison using lDDT as ground truth on CASP16 and CASP15 benchmarks, sorted in increasing order of global Pearson's r (CASP16). Values in bold indicate the best performance.

Type	Method	CASP16					CASP15				
		Global		Per-target average			Global		Per-target average		
		$r\uparrow$	$\rho\uparrow$	$r\uparrow$	$\rho\uparrow$	Loss $\downarrow$	$r\uparrow$	$\rho\uparrow$	$r\uparrow$	$\rho\uparrow$	Loss $\downarrow$
Single-Model	RASP	0.37	0.36	0.50	0.63	0.25	0.55	0.57	0.59	0.55	0.13
	cgRNAsp	0.41	0.41	0.50	0.57	0.09	0.57	0.55	0.64	0.51	0.08
	DFIRE-RNA	0.43	0.43	0.49	0.61	0.09	0.67	0.64	0.72	0.61	0.13
	rsRNAsp1	0.44	0.45	0.53	0.63	0.11	0.61	0.58	0.72	0.66	0.04
	ARES	0.46	0.55	0.48	0.60	0.20	0.52	0.46	0.60	0.56	0.12
	rsRNAsp	0.50	0.54	0.55	0.62	0.13	0.65	0.65	0.72	0.62	0.08
	RNA3DCNN	0.53	0.50	0.68	0.73	0.07	0.43	0.48	0.46	0.42	0.09
	RNArank	0.57	0.66	0.58	0.58	0.09	0.45	0.46	0.57	0.52	0.06
	lociPARSE	0.60	0.69	0.62	0.69	0.07	0.74	0.72	0.73	0.66	0.08
Multi-Model	RNAtive	0.47	0.68	0.49	0.71	0.13	0.67	0.53	0.71	0.77	0.16
	PARSEbp	**0.74**	**0.82**	**0.71**	**0.78**	**0.05**	**0.84**	**0.79**	**0.89**	**0.86**	**0.03**

1.7 Performance w.r.t. TM-score as ground truth

Table S7: Performance comparison using TM-score as ground truth on CASP16 and CASP15 benchmarks, sorted in increasing order of global Pearson's r (CASP16). Values in bold indicate the best performance.

Type	Method	CASP16					CASP15				
		Global		Per-target average			Global		Per-target average		
		$r\uparrow$	$\rho\uparrow$	$r\uparrow$	$\rho\uparrow$	Loss $\downarrow$	$r\uparrow$	$\rho\uparrow$	$r\uparrow$	$\rho\uparrow$	Loss $\downarrow$
Single-Model	RASP	0.39	0.34	0.50	0.52	0.26	0.34	0.40	0.44	0.39	0.16
	RNA3DCNN	0.48	0.47	0.53	0.54	0.14	0.32	0.36	0.34	0.27	0.20
	ARES	0.49	0.46	0.57	0.52	0.24	0.26	0.25	0.45	0.40	0.19
	cgRNAsp	0.52	0.46	0.54	0.51	0.16	0.54	0.54	0.53	0.39	0.16
	DFIRE-RNA	0.53	0.46	0.56	0.53	0.14	0.56	0.55	0.57	0.44	0.21
	rsRNAsp1	0.53	0.46	0.59	0.57	0.16	0.52	0.50	0.60	0.52	0.11
	rsRNAsp	0.56	0.50	0.61	0.57	0.20	**0.65**	**0.67**	0.61	0.50	0.16
	lociPARSE	0.64	0.61	0.66	0.59	0.14	0.56	0.55	0.62	0.54	0.13
	RNArank	0.65	0.70	0.61	0.52	0.14	0.51	0.56	0.62	0.51	0.12
Multi-Model	RNAtive	0.45	0.53	0.61	0.63	0.16	0.29	0.26	0.45	0.53	0.25
	PARSEbp	**0.81**	**0.77**	**0.84**	**0.76**	**0.08**	0.61	0.64	**0.71**	**0.71**	**0.09**

1.8 Performance w.r.t. RMSD as ground truth

Table S8: Performance comparison using all-atom RMSD as ground truth on CASP16 and CASP15 benchmarks, sorted in increasing order of global Pearson's r (CASP16). Values in bold indicate the best performance.

Type	Method	CASP16					CASP15				
		Global		Per-target average			Global		Per-target average		
		$r\uparrow$	$\rho\uparrow$	$r\uparrow$	$\rho\uparrow$	Loss $\downarrow$	$r\uparrow$	$\rho\uparrow$	$r\uparrow$	$\rho\uparrow$	Loss $\downarrow$
Single-Model	RASP	0.23	0.14	0.36	0.34	12.16	-0.03	-0.15	0.22	0.20	13.25
	RNA3DCNN	0.25	0.23	0.39	0.36	5.25	-0.07	-0.17	0.19	0.15	13.35
	cgRNAsp	0.31	0.24	0.40	0.36	7.67	0.05	-0.10	0.52	0.31	11.76
	DFIRE-RNA	0.32	0.22	0.42	0.36	7.59	0.18	0.02	0.56	0.37	12.48
	rsRNAsp1	0.33	0.25	0.44	0.40	7.11	0.14	0.00	0.51	0.37	8.21
	rsRNAsp	0.37	0.29	0.46	0.40	9.46	0.05	-0.01	0.56	0.37	10.99
	ARES	0.37	0.34	0.46	0.39	9.99	0.40	0.34	0.42	0.28	11.06
	lociPARSE	0.46	0.45	0.52	0.43	5.58	0.19	0.16	0.38	0.32	10.16
	RNArank	0.52	**0.62**	0.54	0.45	5.96	0.41	0.44	0.52	0.39	6.55
Multi-Model	RNAtive	0.49	0.51	0.53	0.46	7.20	**0.55**	**0.54**	0.56	0.47	12.76
	PARSEbp	**0.62**	**0.62**	**0.69**	**0.59**	**4.72**	0.46	0.49	**0.61**	**0.56**	**4.44**

1.9 Performance w.r.t. GDT_TS as ground truth

Table S9: Performance comparison using GDT_TS as ground truth on CASP16 and CASP15 benchmarks, sorted in increasing order of global Pearson's r (CASP16). Values in bold indicate the best performance.

Type	Method	CASP16					CASP15				
		Global		Per-target average			Global		Per-target average		
		$r\uparrow$	$\rho\uparrow$	$r\uparrow$	$\rho\uparrow$	Loss $\downarrow$	$r\uparrow$	$\rho\uparrow$	$r\uparrow$	$\rho\uparrow$	Loss $\downarrow$
Single-Model	RASP	0.38	0.31	0.52	0.53	0.24	0.27	0.34	0.44	0.46	0.15
	RNA3DCNN	0.49	0.47	0.54	0.55	0.12	0.26	0.32	0.31	0.28	0.16
	ARES	0.50	0.45	0.58	0.51	0.23	0.18	0.17	0.44	0.43	0.20
	DFIRE-RNA	0.52	0.45	0.56	0.53	0.14	0.55	0.56	0.57	0.49	0.17
	rsRNAsp1	0.52	0.45	0.59	0.56	0.15	0.52	0.52	0.60	0.57	**0.08**
	cgRNAsp	0.52	0.45	0.55	0.51	0.16	0.57	0.58	0.53	0.45	0.10
	rsRNAsp	0.57	0.51	0.61	0.56	0.17	**0.69**	**0.72**	0.61	0.55	0.10
	RNArank	0.64	0.68	0.61	0.51	0.12	0.54	0.55	0.61	0.52	0.11
	lociPARSE	0.65	0.61	0.68	0.60	0.13	0.50	0.49	0.60	0.57	0.13
Multi-Model	RNAtive	0.46	0.52	0.61	0.63	0.15	0.25	0.19	0.45	0.57	0.18
	PARSEbp	**0.82**	**0.77**	**0.85**	**0.76**	**0.08**	0.58	0.60	**0.71**	**0.74**	**0.08**

1.10 CASP16 performance using Fisher-z averaged per-target correlations

Table S10: Performance on 25 CASP16 RNA targets (4,750 decoys) evaluated using two ground-truth metrics, composite score (G) and INF-All. The average of the per-target correlations (r_i) is computed using Fisher's z-transformation with target-specific weights $w_i = N_i - 3$, where N_i is the number of decoys for target i, $z_i = \text{arctanh}(r_i)$, $\bar{z} = \frac{\sum_i w_i z_i}{\sum_i w_i}$. The final per-target average correlation is given by $\bar{r} = \tanh(\bar{z})$. Bold values indicate the best performance.

Type	Method	Composite score (G)					INF-All				
		Global		Per-target average			Global		Per-target average		
		$r \uparrow$	$\rho \uparrow$	$r \uparrow$	$\rho \uparrow$	Loss $\downarrow$	$r \uparrow$	$\rho \uparrow$	$r \uparrow$	$\rho \uparrow$	Loss $\downarrow$
Single-Model	RASP	0.43	0.37	0.56	0.62	0.24	0.60	0.47	0.73	0.69	0.10
	cgRNASp	0.52	0.47	0.61	0.60	0.12	0.61	0.54	0.71	0.68	0.05
	ARES	0.52	0.51	0.59	0.60	0.21	0.65	0.62	0.73	0.68	0.10
	DFIRE-RNA	0.54	0.48	0.61	0.62	0.11	0.66	0.56	0.74	0.70	0.05
	rsRNASp1	0.54	0.48	0.66	0.66	0.12	0.64	0.56	0.76	0.74	0.05
	RNA3DCNN	0.58	0.54	0.71	0.71	0.09	0.61	0.59	0.70	0.72	0.04
	rsRNASp	0.60	0.55	0.67	0.65	0.15	0.64	0.56	0.76	0.73	0.09
	RNArank	0.65	0.68	0.71	0.63	0.10	0.57	0.62	0.65	0.60	0.06
	lociPARSE	0.69	0.67	0.74	0.70	0.09	0.77	0.74	0.81	0.76	0.04
Multi-Model	RNAtive	0.50	0.60	0.65	0.73	0.13	0.59	0.65	0.86	0.83	0.13
	PARSEbp w/o bp†	0.84	0.83	0.88	0.84	**0.05**	0.78	0.78	0.81	0.77	**0.03**
	PARSEbp	**0.86**	**0.84**	**0.90**	**0.86**	**0.05**	**0.85**	**0.83**	**0.88**	**0.84**	**0.03**

† A baseline variant of PARSEbp without the emphasis on base-pairings.

2 Supplementary Figures

2.1 Runtime comparison with SOTA methods

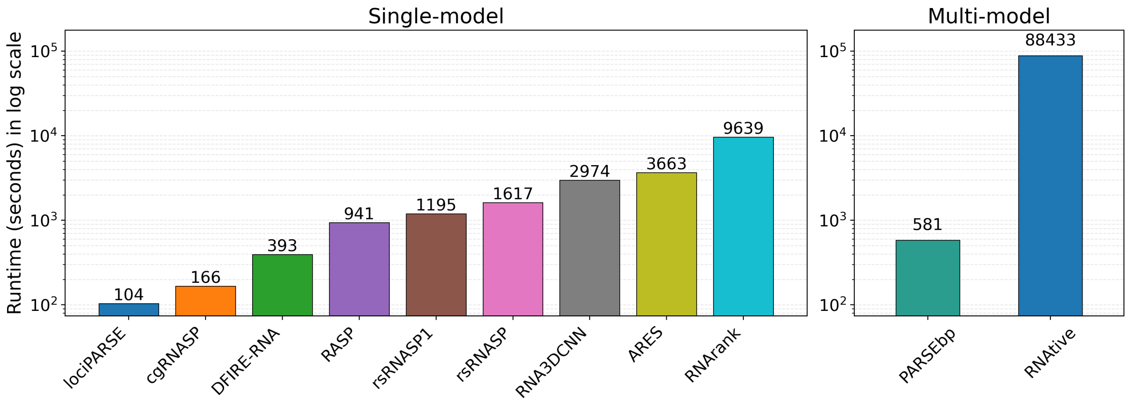

Figure S1: Bar chart showing the runtime comparison of PARSEbp against state-of-the-art methods, plotted on a logarithmic y-axis. Wall-clock runtime (seconds) annotated above each bar denotes the time required by each method to score an ensemble of 1,000 3D structures sampled by RNAbpFlow [1] for a CASP16 target (R1255) with sequence length of 124 nucleotides. Despite being a multi-model QA method, PARSEbp exhibits runtime comparable to several single-model approaches, whereas RNAtive [2] requires substantially longer runtime for large structure pools.

2.2 Runtime requirement of PARSEbp

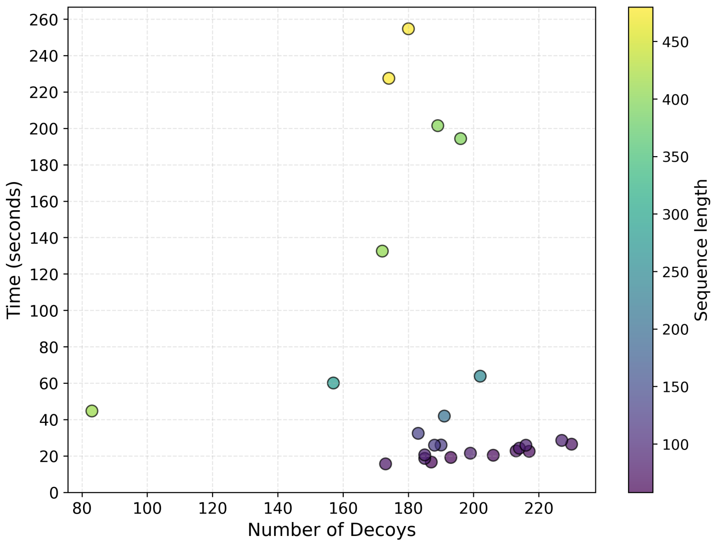

Figure S2: Scatter plot of number of decoys versus runtime (seconds) required for 25 CASP16 targets where the color ramp indicates the increase in sequence length. For typical RNA targets with sequence length of $\sim$ 100 nucleotides, the runtime is very low (approximately 25-35 seconds) with 50 parallel threads, while longer sequences with larger decoy sets naturally require more time.

References

[1] Sumit Tarafder and Debswapna Bhattacharya. RNAbpFlow: Base pair-augmented SE (3)-flow matching for conditional RNA 3D structure generation. *bioRxiv*, 2025.

[2] Jan Pielesiak, Maciej Antczak, Marta Szachniuk, and Tomasz Zok. RNAtive to recognize native-like structure in a set of RNA 3D models. *Bioinformatics*, 41(11):btaf601, 2025.
